# Catheter ablation of a latent accessory pathway under continuous infusion of adenosine: A case report

**DOI:** 10.1097/MD.0000000000021482

**Published:** 2020-07-31

**Authors:** Gabriel Cismaru, Radu Rosu, Mihai Puiu, Gabriel Gusetu, Sabina Istratoaie, Andrei Cismaru, Dana Pop, Dumitru Zdrenghea

**Affiliations:** a Fifth Department of Internal Medicine, Cardiology-Rehabilitation; b Department of Pharmacology, Toxicology and Clinical Pharmacology; c Research Centre for functional Genomics, Biomedicine, and Translational Medicine, Iuliu Hatieganu University of Medicine and Pharmacy, Cluj-Napoca, Romania.

**Keywords:** accessory pathway, adenosine, catheter ablation, left lateral

## Abstract

**Rationale:**

In absence of conduction over the accessory pathway (AP) during the electrophysiological study, mapping and ablation is impossible. Various techniques can be used to activate absent conduction. In this presentation we describe the first case of latent AP ablation performed under continuous infusion of adenosine.

**Patient concerns:**

A 65-year-old man, presented to emergency department with atrial fibrillation and antegrade conduction through a left lateral AP. He had palpitations and lightheadedness that occurred every 2 to 3 weeks.

**Diagnosis:**

The electrophysiological study confirmed a latent left-side AP.

**Interventions:**

Catheter ablation could not be performed because of absent conduction through AP. Therefore, a continuous infusion of adenosine was used to activate AP. Ablation was performed at the left lateral mitral ring.

**Outcomes:**

After catheter ablation and a new adenosine bolus there was no conduction through AP.

**Lessons:**

In case of a latent AP when ablation is difficult to perform because of absent conduction at the time of electrophysiological study, adenosine can be used in doses of 1.5 mg/kg over 5 minutes continuous infusion.

## 1 Introduction

Latent preexcitation is defined as presence of ventricular preexcitation during atrial pacing, atrial extrastimuli or atrial arrhythmias but absent during normal sinus rhythm.^[1]^ However, intermittent preexcitation is characterized by the intermittent presence of preexcited QRS complexes during sinus rhythm with loss of preexcitation during prolongation of the PR interval.^[2]^ In case of latent preexcitation, the conduction time between the sinus node and the ventricle is faster than over the accessory pathway(AP), therefore the delta wave and the short PR interval are absent.

Latent preexcitation is especially observed in cases with a left free-wall accessory pathway and in the presence of an enhanced atrio-ventricular (AV) nodal conduction. For reasons yet unknown, APs may have absent conduction during electrophysiological study, which render mapping and ablation impossible. Among the various techniques used to activate absent conduction, there are: boluses of adenosine, selective site pacing, his-refractory premature ventricular contractions. However, these techniques sometimes fail to yield a successful outcome.

We present the first case of latent AP in which catheter ablation was performed under a continuous infusion of adenosine.

## 2 Case report

A 65-year old male patient with atrial fibrillation conducted anterogradely through an AP, was hospitalized for catheter ablation. He presented recurring episodes of palpitations lasting minutes to hours every 2 to 3 weeks. The physical examination revealed a grade II apical systolic murmur and was otherwise normal, without any sign of left or right heart failure. The twelve-lead ECG showed sinus rhythm with a heart rate of 70 beats per minute, normal PR interval(160ms) with no delta wave. Echocardiography revealed a non-dilated left ventricle with a normal ejection fraction of 58%. Neither the 24-hour Holter ECG, nor the exercise stress test revealed presence of AP – the PR interval was normal and no delta wave was detected. Most probably AP has become latent due to chronic amiodarone treatment of 200 mg/d for several months.

**Figure 1:**
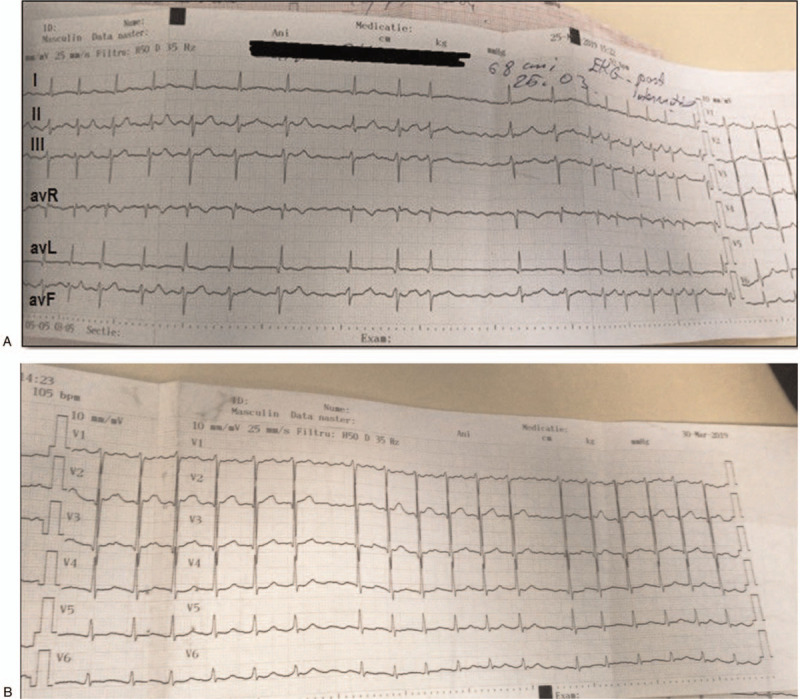
Twelve lead ECG during atrial fibrillation shows narrow QRS without any preexcited beat.

**Figure 2:**
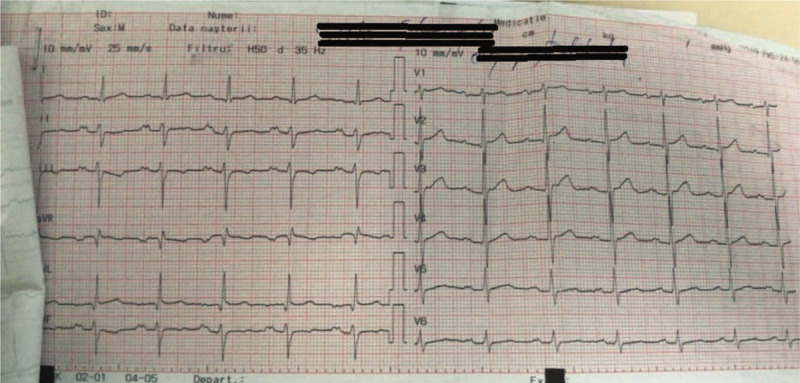
Twelve lead ECG during sinus rhythm shows narrow QRS without any preexcited beat.

**Figure 3:**
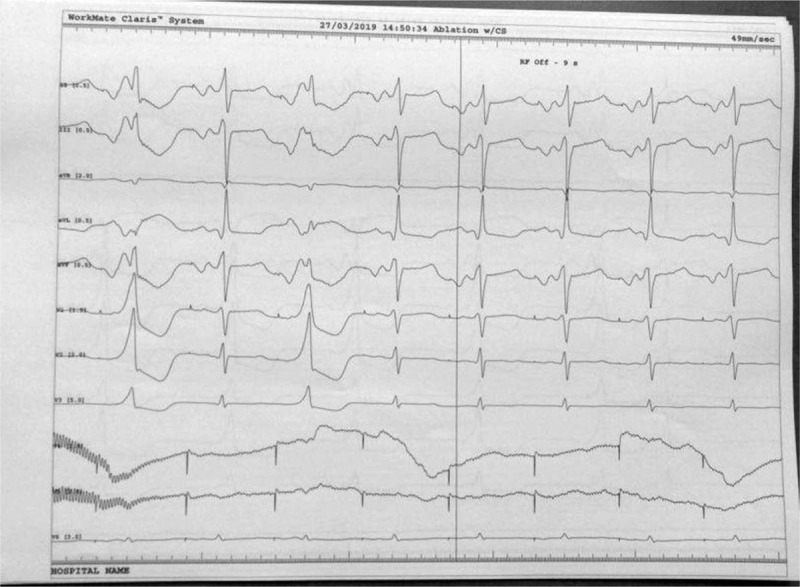
During electrophysiological study we induced atrial fibrillation. There is no preexcited beat.

**Figure 4:**
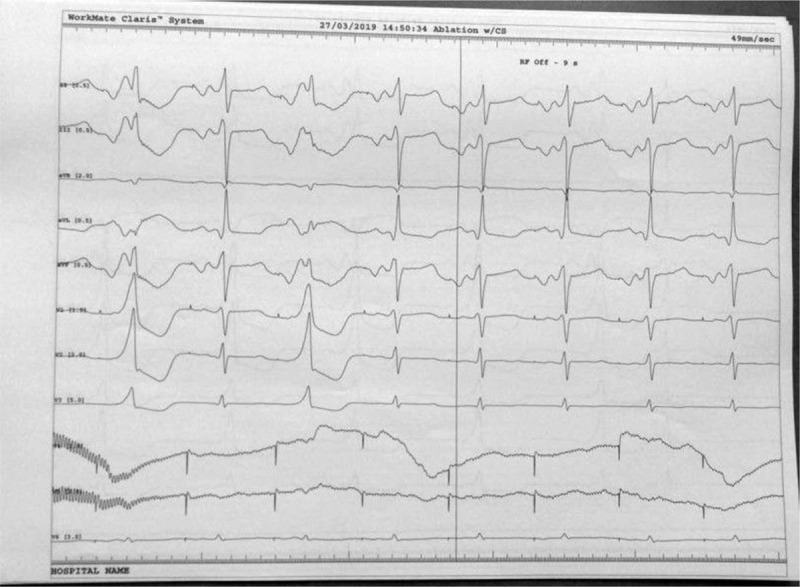
After a bolus of 18 mg of adenosine only some beats are preexcited during atrial stimulation, making the mapping and ablation impossible.

**Figure 5:**
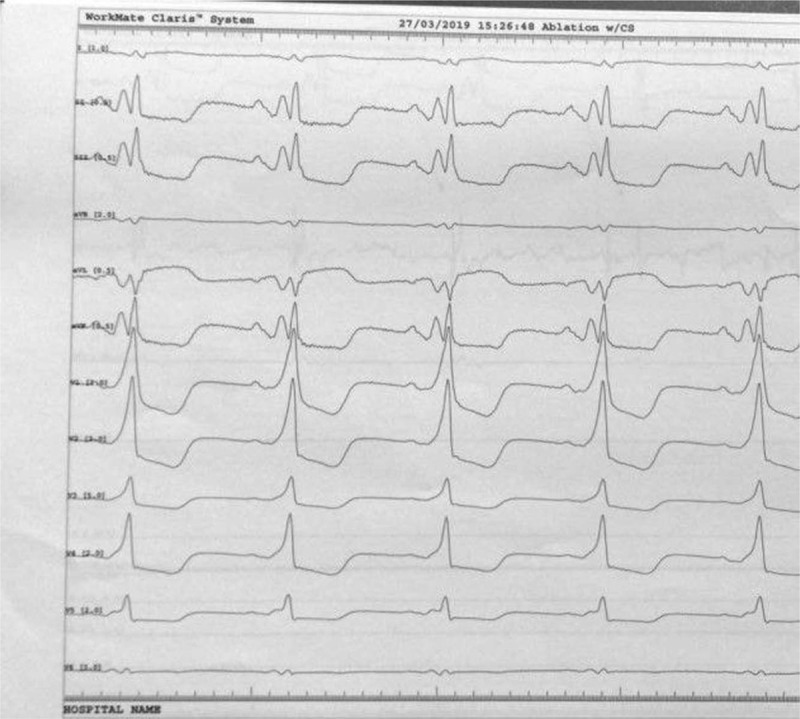
During continuous infusion of 1.5 mg/kg adenosine over 5 minutes the accessory pathway is present, allowing mapping at the level of lateral mitral ring.

**Figure 6:**
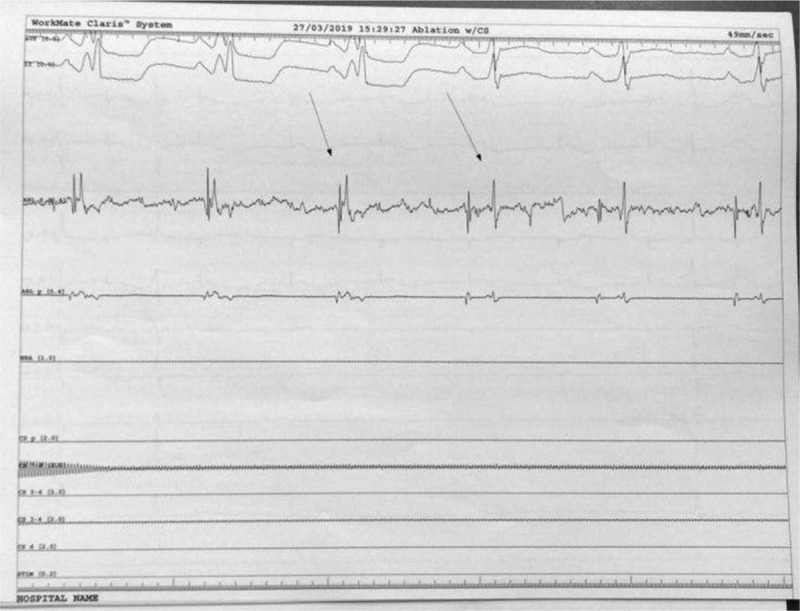
Catheter ablation of the accessory pathway: the arrow shows the local potential at the level of the left lateral mitral ring before and after ablation. Please note the short AV interval before accessory pathway ablation that comes to normal after ablation.

**Figure 7:**
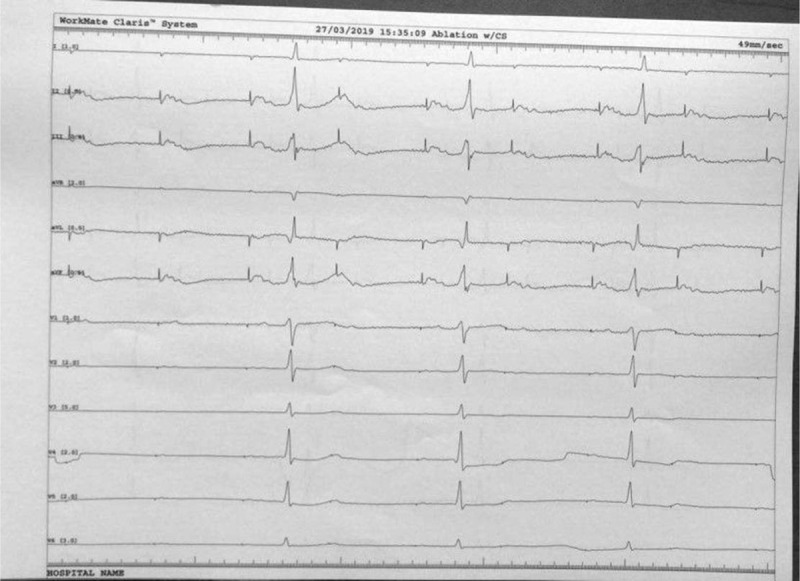
Twelve-lead electrocardiogram during Adenosine infusion after catheter ablation of the accessory pathway shows complete AV block and 2:1 AV block during atrial fixed stimulation.

**Figure 8:**
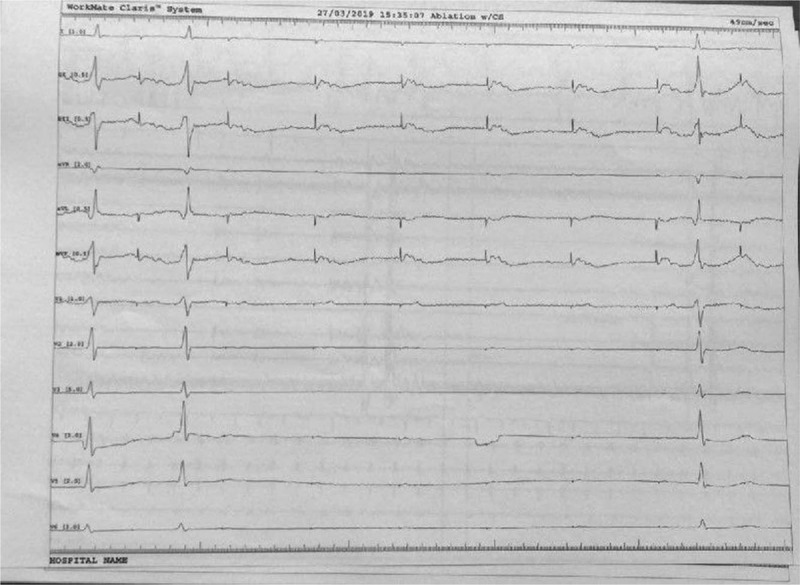
Twelve-lead ECG after 18 mg bolus of Adenosine during atrial stimulation shows complete AV block after catheter ablation of the accessory pathway.

The patient underwent an electrophysiological study under local anesthesia. During hospitalization, the ECG records in both atrial fibrillation and sinus rhythm revealed no AP (Figure 1A,1 B,2). A catheter was placed in each of the: coronary sinus, right ventricle, high right atrium, and at the His bundle region. Programmed stimulation of the atria and ventricles was performed. A normal His-ventricle (HV) interval of 48 ms was documented without any sign of antegrade or retrograde conduction through an AP. After atropine injection repeated atrial and ventricular stimulation could not induce any reentrant tachycardia and the HV remained normal with antegrade and retrograde conduction through the nodal pathway. Atrial fibrillation was induced by aggressive atrial stimulation of 150 ms but it only resulted in narrow QRS and no conduction through an AP (Fig. 3). As a result, we decided to inject adenosine boluses of 12, 18, and 36 mg followed by 10 cc of saline flush, with 3 to 5 beats conducted through a left lateral AP (Fig. 4). Mapping could not be performed during the transient effect of adenosine. Since 5 mg of betablocker and 1 vial of Digoxin were injected but without any sign of AP, the AP was further mapped under continuous adenosine infusion of 1.5 mg/kg adenosine over 5 minutes. Under adenosine infusion the AP became apparent (Fig. 5) and the mapping of the lateral mitral ring together with catheter ablation using the transseptal approach were possible. Only antegrade conduction through the AP was observed, without retrograde conduction. After 3 minutes of mapping, a good spot was found in the left lateral wall and catheter ablation was performed (Fig. 6) with subsequent complete AV block due to adenosine (Fig. 7). After the infusion was stopped the normal sinus rhythm resumed. Upon repeating the bolus of adenosine with complete AV block no sign of AP was detected (Fig. 8). Programmed atrial and ventricular stimulation was performed without conduction through an AP.

In the follow-up, a catheter ablation of atrial fibrillation was also performed as the patient continued to present atrial fibrillation (AF) with narrow QRS.

## 3 Discussion

Adenosine infusion is currently used in cardiology to study the coronary vasodilator reserve,^[3]^ to reverse pulmonary vasoconstriction,^[4]^ to assess fractional flow reserve (FFR) and to evaluate the myocardium during pharmacological myocardial stress test.^[5]^

High dose adenosine infusion of 200 μg/kg over 3-minute through the femoral vein was used by Alexopoulos et al in a prospective study on 30 consecutive patients with significant coronary stenosis assessed by FFR. Discontinuation of the infusion was not required in any patient and the majority of them reported minor side effects like flushing, dyspnea, and chest discomfort. Seven patients (23.3%) presented transient second or third degree AV block.^[6]^ Doses up to 210 μg/kg/mn are used in interventional cardiology for FFR measurement.^[7]^ Our patient received 1.5 mg/kg adenosine and presented flushing during the first 30 seconds, without any further side-effects.

Adenosine can be used in electrophysiological study to reveal AP conduction in case of latent preexcitation.^[1]^ Latent preexcitation is defined as absence of ventricular preexcitation during sinus rhythm with presence during atrial pacing or premature atrial contractions or other atrial arrhythmias. Intermittent preexcitation is a term that defines presence of preexcited complexes intermittently during sinus rhythm and loss of preexcitation when prolongation of the PR interval occurs.^[2]^ In some patients with APs preexcitation occurs intermittently during sinus rhythm. In these patients the antegrade refractory period of the AP may either exceed the sinus cycle length under some circumstances, or conduction block in the AP may be variable. These phenomena probably reflect variations in the autonomic tone.

It is well known that amiodarone in patients with ventricular preexcitation can normalize the shortened PR interval and make delta wave disappear^[8]^ In order to perform catheter ablation, there must be evidence of the presence of the AP during the electrophysiological study. The ability of adenosine to unmask latent preexcitation was demonstrated by Morgan–Hughes et al on 6 patients that received incremental doses of adenosine 3.6 and 12 mg. This procedure revealed preexcitation in all patients. The authors explain the effect of adenosine by 2 mechanisms: a direct inhibitory effect on the AV node and an enhanced conduction through the AP due to increased sympathetic activity.^[1]^ However, the short half-life of adenosine has made assessment of its effects on antegrade refractoriness of APs difficult. Therefore, we continuously injected adenosine in our patient.

The dose of adenosine used in our study was 1.5 mg/kg over 5 minutes which corresponds to 90 mg infused. To date, there is no medical data that shows adverse effects related to a high cumulative dose of intravenous adenosine. Lapage et al used boluses of adenosine to activate conduction through the AP in 7 patients with absent AP conduction during electrophysiological study. Repeated boluses of 12 mg of adenosine were used and there was no superior limit for the cumulative dose of adenosine. Authors used 11.7 vials/patient with a range of 6 to 20 vials corresponding to 1.82 mg/kg (range 1.3 to 2.3 mg/kg). No patient manifested adverse effects to adenosine.

Similar to the study of Lapage et al other options were explored to unveil the AP before adenosine.^[9]^ While Lapage et al used esmolol, phenylephrine and isoproterenol, which were ineffective in enhancing conduction through the AP, we injected Betaloc 10 mg and 0.5 mg of Digoxin without any visible effect on the AP.

## 4 Conclusions

In case of a latent AP, ablation can be difficult because of absent conduction at the time of electrophysiological study.

Continuous adenosine infusion in doses of 1.5 mg/kg over 5 minutes is safe and can be used to make the mapping of the AP possible.

### 4.1 Consent

Informed written consent was obtained from the patient for the publication of this case report with accompanying medical images.

## Author contributions

**Conceptualization:** Gabriel Cismaru, Gabriel Gusetu, Sabina Istratoaie, Dana Pop.

**Formal analysis:** Gabriel Cismaru.

**Investigation:** Gabriel Cismaru, Rosu Radu, Mihai Puiu, Gabriel Gusetu, Sabina Istratoaie, Dumitru Zdrenghea.

**Methodology:** Gabriel Cismaru, Rosu Radu, Mihai Puiu, Gabriel Gusetu, Sabina Istratoaie, Dana Pop.

**Supervision:** Gabriel Cismaru, Rosu Radu, Dana Pop, Dumitru Zdrenghea.

**Validation:** Gabriel Cismaru, Gabriel Gusetu, Dana Pop, Dumitru Zdrenghea.

**Visualization:** Gabriel Cismaru, Mihai Puiu, Sabina Istratoaie, Dana Pop, Dumitru Zdrenghea.

**Writing – original draft:** Gabriel Cismaru, Gabriel Gusetu, Sabina Istratoaie.

**Writing – review & editing:** Gabriel Cismaru, Dana Pop, Dumitru Zdrenghea.
